# Bioactive metabolites from marine fungal sources: evaluation of antimicrobial and cytotoxic activities

**DOI:** 10.1186/s12866-026-04962-4

**Published:** 2026-04-06

**Authors:** Ahmed A. Hamed, Mohamed E. Elawady, Mohamed H. Yassin, Dina. M.M. Elnagar, Mervat G. Hassan

**Affiliations:** 1 Microbial Chemistry Department, National Research Centre, El-Buhouth Street, Dokki, Cairo, Egypt; 2 Microbial Biotechnology Department, National Research Centre, El- Buhouth Street, Dokki, Cairo, Egypt; 3 Microbiology Department, Faculty of Science, Benha University, Benha, Egypt

**Keywords:** Marine fungi, *Penicillium rubens*, GC-MS analysis, Antibacterial activity, Cytotoxicity, ADME prediction

## Abstract

Marine associated fungi were isolated from marine sponges collected at two ecologically distinct localities in Hurghada. Macroscopic characteristics were employed to identify and classify distinct fungal strains using ethanol-hypochlorite surface sterilization. The fungal isolates, identified as marine fungi, were cultivated on Egyptian white rice media to yield bioactive crude extracts following ethyl acetate extraction. The most potent isolate was strain DI7S9. This strain, morphologically and genetically identified as *Penicillium rubens* (GenBank: OP709283.1), was extensively fermented to yield biomass for subsequent experiments. The primary component of the extract was trans-9-octadecenoic acid, pentyl ester, as determined by GC-MS analysis. According to SwissADME, this compound exhibits moderate drug-likeness, limited gastrointestinal absorption, and low permeability across the blood-brain barrier. The crude extracts exhibited selective cytotoxicity, effectively inhibiting malignant HCT-116 and A-498 cells at low IC₅₀ values (30.44 and 37.99 µg/mL), while inflicting minimal damage on normal HEK-293 cells (IC₅₀ = 77.89 µg/mL). Notable suppression of *E. coli*, *S. aureus*,* A. niger*, and *C. albicans* was observed. The metabolites of P. rubens DI7S9 exhibit significant pharmacological potential, warranting further investigation into their mechanisms of action and potential therapeutic applications.

**Graphical Abstract:**

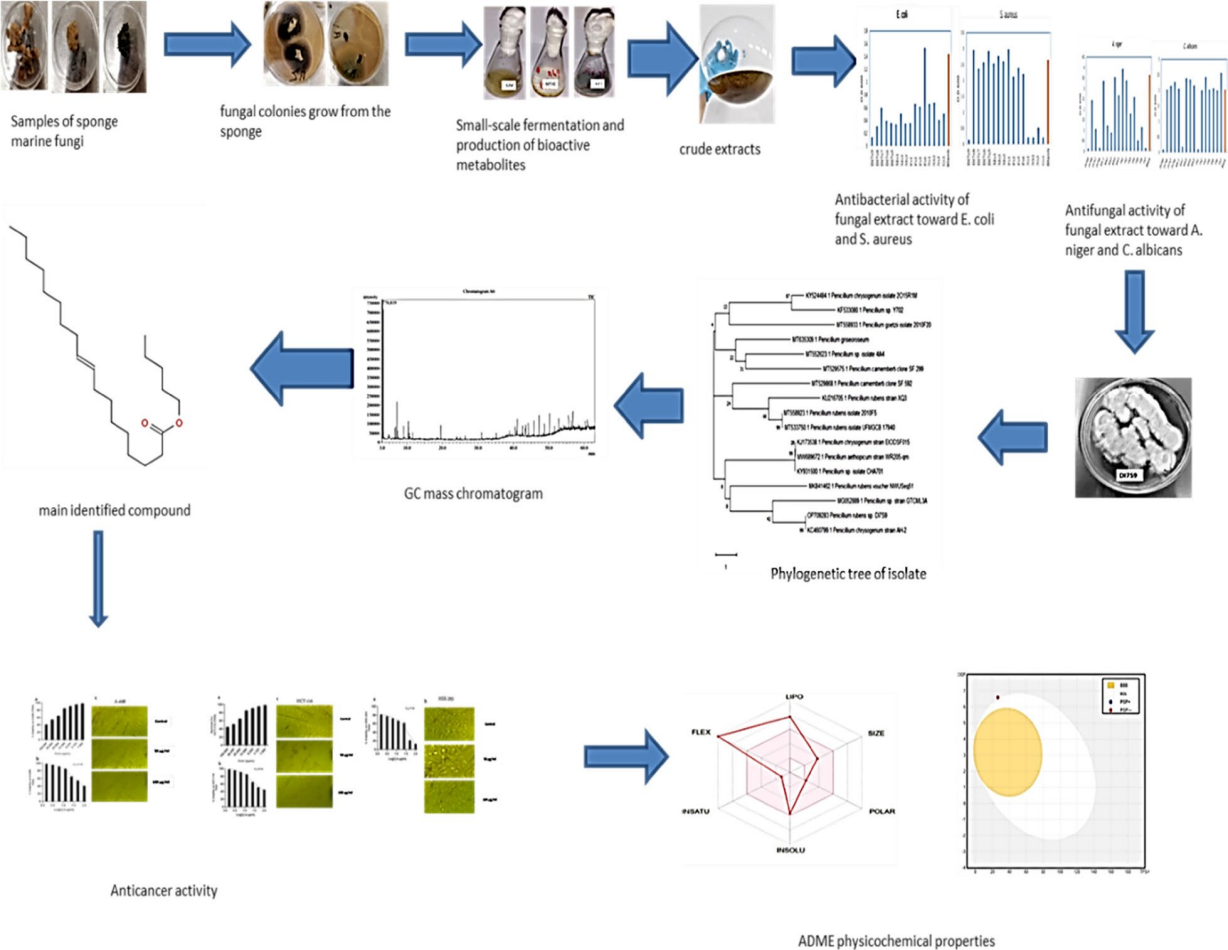

**Supplementary Information:**

The online version contains supplementary material available at 10.1186/s12866-026-04962-4.

## Introduction

Natural products are naturally occurring substances that do not directly participate in an organism’s typical growth, development, or reproduction [1]. Natural ingredients have been utilized since antiquity and in folklore to treat many diseases and ailments. Using traditional methods of natural product chemistry, numerous advantageous metabolites from both marine and terrestrial sources were discovered. Numerous natural compounds have evolved into contemporary medication possibilities. Several reviews describes the historical significant bioactive marine and terrestrial natural compounds. Marine and terrestrial fungi are abundant producers of these chemicals, and fungal communities’ roles in natural product synthesis are highlighted. We cover traditional uses of microbially generated compounds and how to accelerate their identification and characterization [2]. Chemotherapy is a primary treatment employed to address cancer. Many antitumor agents are natural chemicals or their derivatives, primarily synthesized by marine fungi. Fungi are primarily responsible for the synthesis of numerous natural compounds that exhibit diverse biological activities, including anticancer properties. These anticancer drugs are classified into structural classes, including non-ribosomal peptides, macrolides, isoprenoids, indolocarbazoles, enediynes, and anthracyclines. They fight tumors by inducing apoptosis through DNA cleavage, which is aided by inhibiting topoisomerase I or II, mitochondrial permeabilization, vital enzymes that control signal transduction, such as proteases, or cell metabolism, and, in some cases, tumor-induced angiogenesis. Medicinal substances originating from marine species have garnered significant attention recently due to their potential applications in medicine [3]. The scientific community has given natural products (NPs) a great deal of attention in recent decades, and this interest is only growing. Marine fungi are considered a significant and promising source for production of numerous new chemicals for use in the pharmaceutical, agricultural, and other industries [4]. Among sponge-associated microorganisms, fungi represent an important group inhabiting the tissues of marine sponges produce essential secondary metabolites that support the host growth and increase the host resistance to biotic and abiotic stresses [5–7]. Marine fungi generate a diverse spectrum of metabolites from several chemical classes, including alkaloids, flavonoids, steroids, terpenoids, and phenolic chemicals. Certain compounds exhibit pleiotropic and notable pharmacological activity, including antibacterial, antioxidant, anti-diabetic, antimalarial, and anticancer activities. Identifying these structurally distinct and changeable active chemicals is an important resource for researching natural medicinal agents produced by fungal communities [8–10].

Currently, approximately 5% of the 1.5 million fungus species on Earth have been thoroughly documented; only 16% (11,500) of the 69,000 species in this category have been cultivated and studied. Next-generation sequencing technologies have found approximately 0.035 to 5.1 million fungus species on Earth [11].

This study aimed to enhance our understanding of the biological activities of metabolites produced by marine sponge-associated fungi, particularly their potential as antibacterial agents. Marine fungi inhabiting marine sponges are recognized as important sources of bioactive compounds. The findings of this study may contribute to elucidating the bioactive potential of these fungi and may provide a scientific basis for the development of novel antibacterial agents.

## Materials and methods

### Sample collection

Six Marine sponges were collected in two locations in Hurghada, Abu Minqar Island, a coastal city famed for its aquatic environments: Location 1 and Location 2 (27.25° N, 33.81° E). Since sponges are known to possess bioactive compounds with medicinal potential, they were chosen. SCUBA diving was used to collect sponge samples.

### Isolation of marine fungi associated with marine sponges

The marine associated fungi were isolated using surface sterilization techniques on PDA medium (1 L) with potato extract (4 g), dextrose (20 g), sea salt (24.4 g), agar (20 g), and distilled water inoculated sponge samples with fungus. The pH of the medium was adjusted to 6.0. After surface sterilization, sponges were gently rinsed with tap water and separated. The sponges were cleaned thrice in sterile distilled water after a minute in 70% ethanol and 2% sodium hypochlorite. After sterilization, the sponges were air-dried inside the laminar cabinet. Small pieces of dried sponge, filter-sterilized chloramphenicol (200 mg/L), and nalidixic acid (50 mg/L) were added to the PDA medium after sterilization to limit bacteria growth. For fungal growth, the plates were incubated at 28 degrees Celsius. Several subcultures of chosen fungal colonies resulted in pure colonies. The Microbial Chemistry Department of the National Research Centre (NRC) maintained pure fungal isolates in glycerol 50% at -20 °C [6, 12–14].

### Genotypic identification of fungal isolates

#### DNA extraction

The fungal isolates were identified by 18 S rDNA sequencing. To extract fungal DNA, mycelia were put in a 250 mL Erlenmeyer flask with 50 mL potato dextrose broth. The mixture was then incubated at 28 °C for four days. After incubation, genomic DNA was isolated from mycelial biomass using the Qiagen DNeasy Mini Kit, USA, per the manufacturer’s instructions [15].

#### PCR amplification

NS3 and NS4 universal primers amplified 18 S rRNA [16]. PCR’s temperature profile included a 5-minute initial denaturation stage at 94 °C, 35 cycles of 94 °C for 30 s, 55 °C for 30 s, 72 °C for 90 s, and a 5-minute final extension step at 72 °C.

#### Sequencing

The amplified products were examined by electrophoresis and sequenced in Macrogen Companies, South Korea. The sequence produced was analyzed by using the BLASTN program to study the similarity and homology of the 18 S rRNA gene sequences with the similar existing sequences available in the NCBI database as detected [17].

#### Large-scale fermentation, extraction

*Penicillium rubens* DI7S9, the most potent fungal isolate, was cultivated in a 10-L rice-solid medium for large-scale fermentation. The medium consisted of 100 g of commercially available Egyptian white rice and 100 mL of 50% (v/v) naturally salinized artificial seawater. After incubation for 15 days at 28 °C, the cultures were extracted with ethyl acetate (EtOAc), and the organic phase was concentrated under vacuum to obtain the crude extract [18–20].

#### GC-mass analysis

For fungal extract analysis, GC (THERMO Scientific Corp., Waltham, MA, USA) and MS were used. Fungal extract (1 µL) was put onto a 30 m × 0.32 mm × 0.25 μm chromatographic column using the Autosampler AS1300. After initial analysis at 60 °C, temperature cycling was raised to 240 °C. Next, it was progressively raised to 290 °C for 2 min. The GC–MS analysis was carried out using an HP-5MS capillary column (Agilent Technologies) with a length of 30 m, an internal diameter of 0.25 mm, and a film thickness of 0.25 μm. The column was coated with a stationary phase composed of 5% phenyl and 95% dimethylpolysiloxane, which provides excellent separation efficiency for a wide range of volatile and semi-volatile organic compounds. High-purity helium was used as a carrier at 1 mL/min. The electron ionization mass of the spectra was taken in full scan mode at m/z 40–1000 using 70 eV electron energy as intended. The new chemicals were identified by comparing their retention time (RT) and mass spectra to those in the National Institute of Standards and Technology collection.

#### Antimicrobial testing

Gram-positive, penicillin-resistant *Staphylococcus aureus*, Gram-negative, sensitive E. coli and fungi (*Candida albicans*,* Aspergillus niger*), were used to test the antibacterial efficacy of fungus crude extracts. Microplate dilution was used in the experiment. In summary, 10 µL of fungal (250 µg/mL) were mixed with 180 µL of potato dextrose broth for fungi and lysogeny broth for bacteria. Next, 10 µL of the bacterial or fungal logarithmic growth phase solution was added to the Spectrostar Nano Microplate Reader (BMG Labtech GmbH). Allmendgrun GmbH in Germany determined the absorbance at OD_600_ following an overnight incubation at 37 °C.

#### Anticancer activity

Anticancer activity was carried out toward different cell lines, including A-498, HCT-116, and HEK-293. A-498 is a human kidney carcinoma cell line derived from renal cancer. HCT-116 is a human colorectal carcinoma cell line extensively used for studying colorectal cancer. HEK-293, derived from human embryonic kidney cells, is commonly employed in molecular biology and biotechnology research. The cell lines were supplemented from Sigma-Aldrich, USA. Cultivation of cancer cells was done at 37 °C in a humid atmosphere with 5% CO₂, 10% FBS, streptomycin (100 µg/mL), and penicillin (100 U/mL).

#### The cytotoxicity test

The 3-(4,5-dimethylthiazol-2-yl)-2,5-diphenyl tetrazolium bromide (MTT) assay was used to measure the cytotoxic activity of the fungal crude extract, which represents bioactive secondary metabolites, in order to evaluate cell survival and morphological changes. A modified Van Loosdrecht method was used to conduct the assay [21]. Propylene glycol (Sigma-Aldrich, USA) was used to dissolve the fungal crude extract. After stock dilution and culture medium correction, the final concentration of propylene glycol in each well was less than 0.1% (v/v). The control group consisted of cells that were only exposed to the vehicle (0.1% propylene glycol).

To enable monolayer development, cells were seeded into 96-well tissue culture plates at a density of 1 × 10⁵ cells/mL and incubated for 24 h at 37 °C. The cell monolayers were twice cleaned with washing buffer once they reached confluence. After that, RPMI medium supplemented with 2% maintenance serum was used to serially dilute the fungal crude extract. As negative controls, three wells with only maintenance medium were added, while the other wells were filled with 0.1 mL of each extract dilution.

Using an ELISA plate reader (BioTek, USA), absorbance at 570 nm was used to measure cell survival following treatment. GraphPad Prism version 8.2.4 was used to analyze the data and compute the half-maximal inhibitory concentration (IC₅₀) values. Throughout the experiment, changes in the physical properties and morphology of the cells were tracked and recorded. Every test was run in triplicate.

### Statistical analysis

All experiments were performed in triplicate, and the results were expressed as mean ± standard deviation (SD). Statistical analysis was carried out using SPSS software (version XX; IBM Corp., Armonk, NY, USA).

## Results

### Marine sample collection

**Fig. 1:**
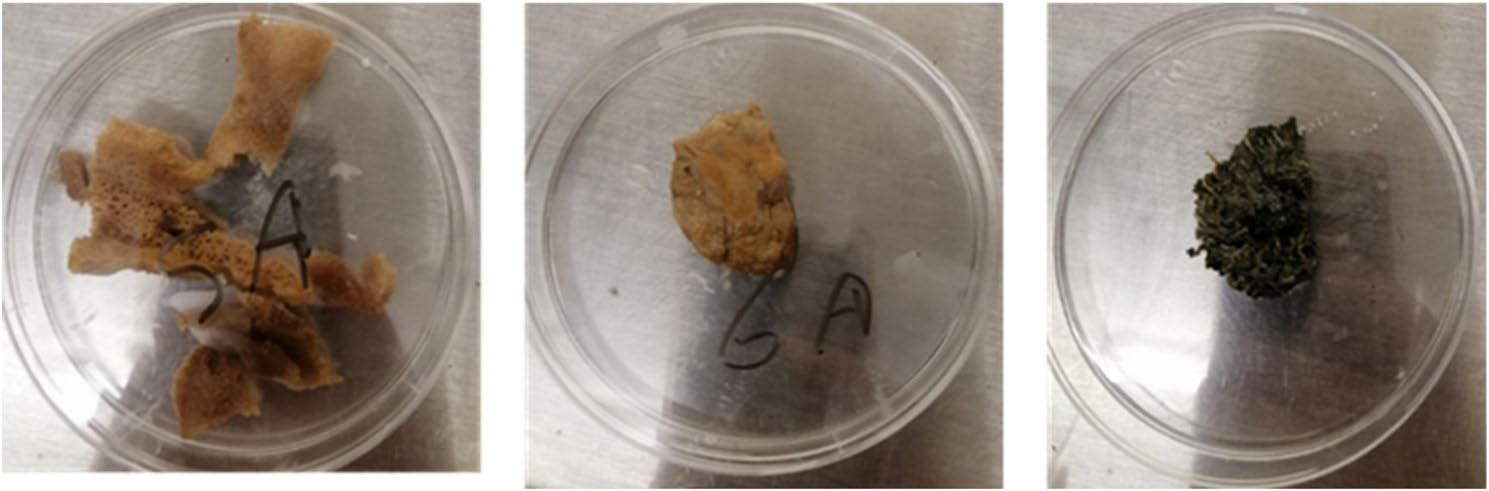
Samples sponge marine fungi

Samples were taken at Location 1 and Location 2, There are two unique places in Hurghada. The gathered samples were carefully moved to the lab for further investigation. As soon as the samples were in the lab, pictures of them were taken to document their physical characteristics and any noticeable variations. After being documented, the samples were stored in the lab for further study. (Supplementary Table 1) compiled and recorded the data, with representative images. The samples are depicted in Fig. 1. The ongoing research relies on these data to accurately document samples from each site.

### Marine fungi isolation

**Fig. 2:**
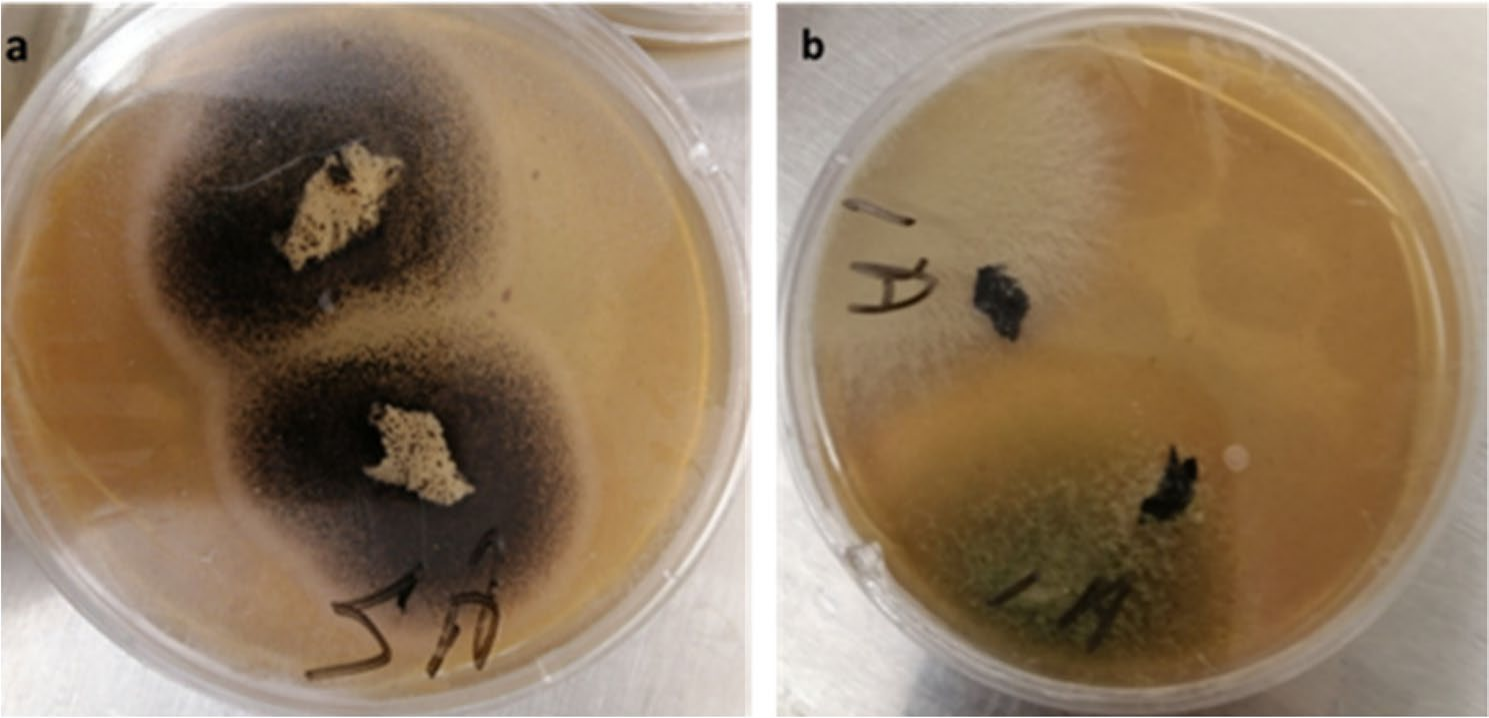
Growth of fungal colonies from sponge sample A5 (**a**) and sample A1 (**b**)

The ethanol and hypochlorite sterility protocol worked well for separating the sponge-associated fungi. Following sterilization, agar plates were used to separate the sponge samples from the fungal colonies, and fungal isolation procedures were applied to obtain pure fungal isolates. The isolated fungal colonies’ color, texture, and growth patterns were examined visually. Morphological features allowed the separated fungi’s taxonomy and group separation. Variations in colony shape revealed sponge-related fungus diversity. These morphologically unique mushrooms are a great starting point for further investigation and description (Table 1; Fig. 2).

**Table 1:** Fungi isolated from various sponges

Marine samples code	Isolated fungus	Morphology
A1	Di7s5	Off-white
	Di7s6	Black
	Di7s7	Green
	Di7s8	Grey
	Di7s9	Yellowish White
A2	Ms1	brown
	Ms2	Green
	Ms3	brown
A3	Fs1	Pale black
	Fs2	Black
A4	Fs5	Pale green
	Fs6	Pale brown
A5	Ns1	Off-white
	Ns2	Black
	Ns3	White
A6	Gs1	black

### Extraction and fermentation on a small scale

**Fig. 3:**
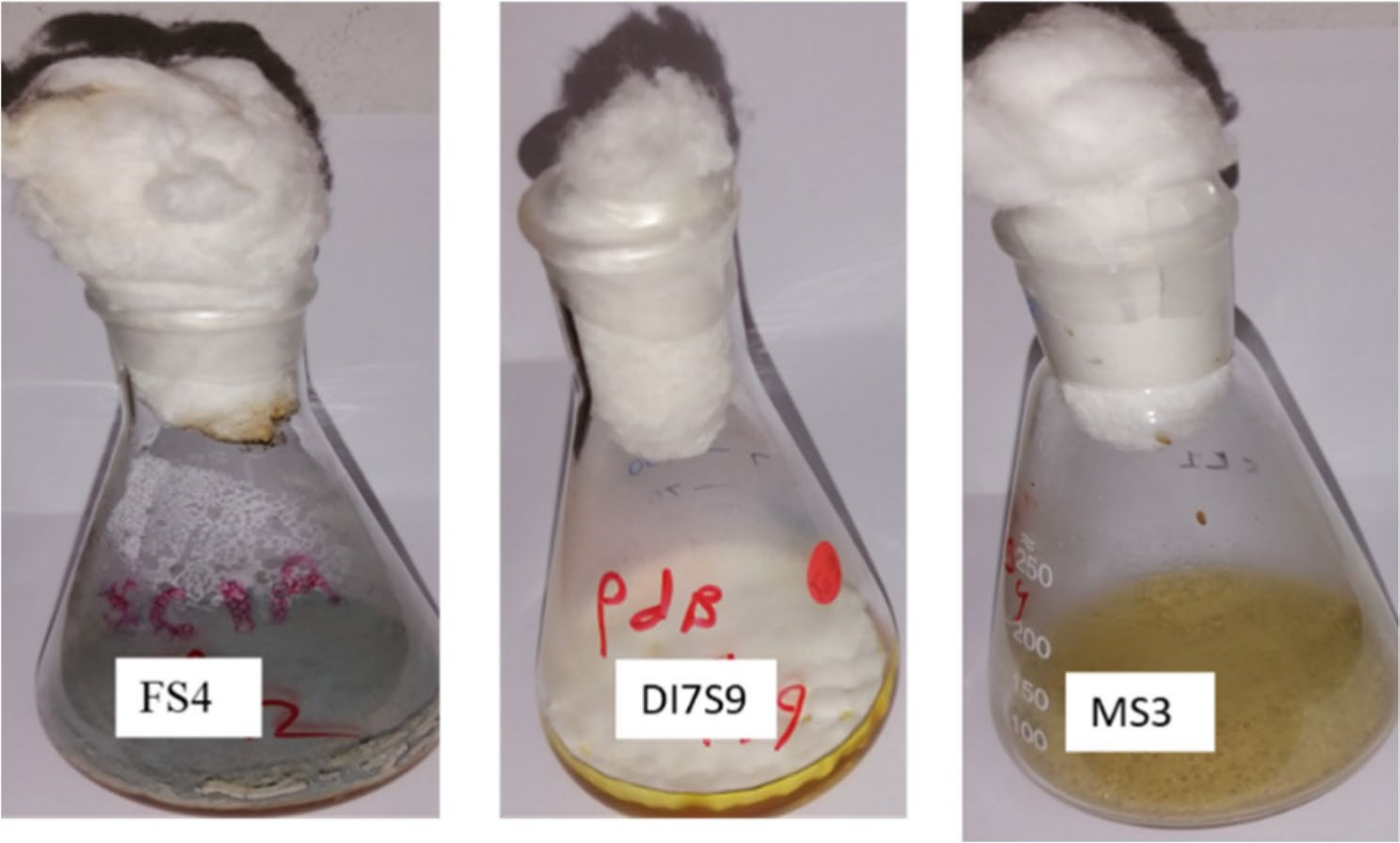
Bioactive metabolite production and small-scale fermentation

The isolated fungal strains were cultivated on a rice-based medium to promote a small-scale fermentation process for the extraction of bioactive components. After adding the fungal spore suspensions, 250 mL Erlenmeyer flasks with 25 g of solid Egyptian white rice substrate each were incubated for 15 days. The generated materials were subsequently extracted using ethyl acetate, as shown in Table 2. The ethyl acetate phase was completely evaporated to dryness upon extraction, indicating that it was suitable for further examination (Table 2. Fig. 3).

**Table 2:** Bioactive compound fermentation and extraction

Isolate Code	Host Organism	Fungal isolate	Obtained weight
1 A	Sponge	DI7S5	1.23
		DI7S6	0.95
		DI7S7	0.72
		DI7S8	1.68
		Di7s9	1.31
2 A	Sponge	MS1	1.50
		MS2	2.30
		MS3	3.2
3 A	Sponge	FS1	1.14
		FS2	1.10
4 A	Sponge	Fs5	2.35
		Fs6	0.80
5 A	Sponge	Ns1	1.03
		NS2	0.72
		Ns3	1.42
6 A	Sponge	Gs1	1.13

### Antibacterial screening of fungal crude extracts

In vitro, crude extracts of sixteen fungal isolates were tested for antibacterial activity against two bacteria, and two fungi. The medicine’s efficacy is demonstrated by its antibacterial activity against *S. aureus* and *E. coli.* Stopping these harmful microorganisms is crucial to preventing nosocomial and foodborne diseases [22–24]. The crude fungal extract with bioactive metabolites may rupture bacterial cell membranes or interfere with critical biological operations, slowing growth and killing cells. The crude extract may also be antifungal because it inhibits *A. niger*, a common food-destroying fungus species. Inhibiting *A. niger’s* growth preserves food quality and prevents foodborne fungus contamination. Understanding its mode of action and ability to damage fungal cell structures or metabolic pathways can improve food preservation and storage. Clinically, the extract’s antifungal action against *Candida albicans*, pathogenic yeast that causes opportunistic infections in humans, is essential. Candida albicans can cause vaginal yeast infections and oral thrush. The chemical’s capacity to prevent yeast growth may affect fungal infection drugs. The fungal crude extract displays antibacterial action against *E. coli*,* S. aureus*,* A. niger*, and *Candida albicans* (Supplementary Fig. 2, 3).

### Morphological identification of the most potent fungal strainDI7S9

**Fig. 4:**
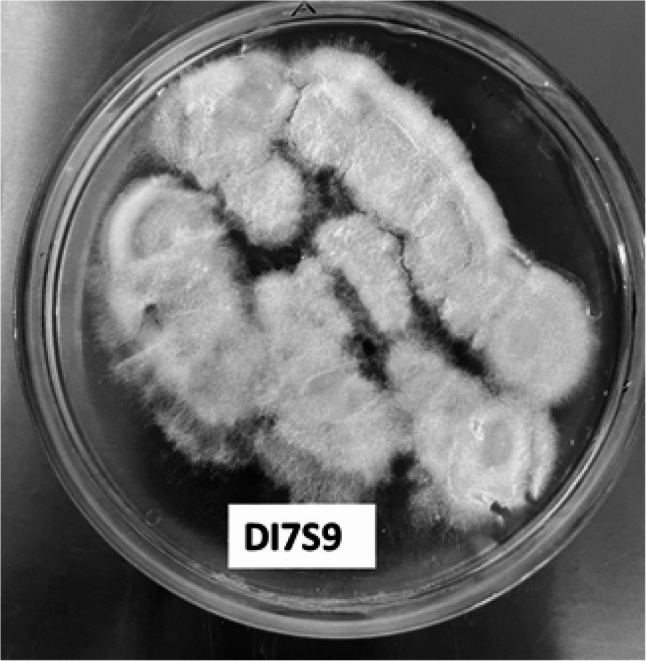
Colony shape and morphology

Morphological identification of the most potent fungal isolate was performed, and the result showed that *Penicillium rubens* DI7S9 has morphological characteristics that are highly variable and dependent on the growth conditions and substrates. When Penicillium *rubens* DI7S9 is grown on a suitable agar medium, it looks colonial. The texture of the colony changes with growth, going from small and cottony to granular or powdery (Fig. 4).

### Genetic identification of the most potent strain DI7S9

The strongest one was genetically determined using the 18SrRNA gene and named DI7S9. The 18 S rRNA gene sequence was retrieved, and it was compared to other sequences in the GenBank database using BLAST (http://www.blast.ncbi.nlm.nih.gov/Blast), allowing the statistically significant differences and similarity scores to be determined. Based on the 18 S rRNA gene sequence, the results demonstrated a high degree of similarity with the *Penicillium rubens* and 100% identity with the DI7S9 isolate.

Saitou and Nei developed a technique called neighbor-joining to trace the evolutionary history [25]. The robustness of the tree’s topology was assessed using a 500-iteration bootstrap test, which showed the frequency with which related taxa clustered near to Felsenstein’s branches [26]. The Maximum Composite Likelihood approach was used to calculate evolutionary distances, which are represented by the number of base substitutions per site [27]. The scale-drawn tree’s branch lengths were utilized to indicate evolutionary distances.

**Fig. 5:**
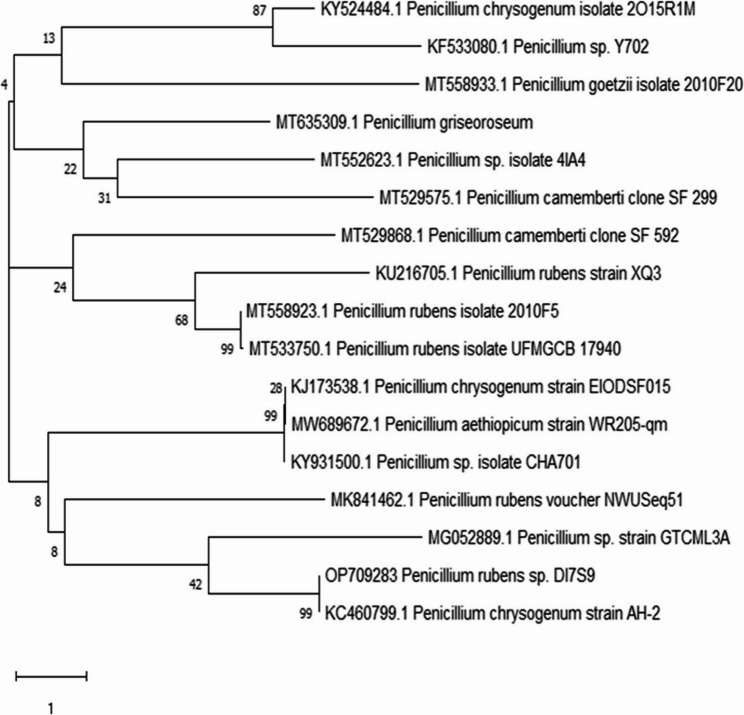
Phylogenetic tree of DI7S9 isolate

This study used a set of fifteen nucleotide sequences representing the first, second, third, and non-coding codon positions. A total of 552 locations were obtained by removing the unclear portions of each sequencing pair (pairwise deletion option). According to the 2018 paper by Kumar [28], evolutionary analysis was carried out using the MEGA X program. The isolate’s morphological characteristics and DNA sequence analysis were used to determine the strain is identical to *Penicillium rubens* DI7S9. The GenBank entry number OP709283.1 for this strain denotes its official database addition.1. (Fig. 5) shows the *Penicillium rubens*DI7S9 strain’s phylogenetic tree.

### Large-scale fermentation, extraction, and bio-guided fractionation

*Penicillium rubens* DI7S9, the most potent strain of the chosen fungus, was grown in a 10-liter rice medium in preparation for large-scale fermentation. Following 15 days of incubation at 28 °C, the medium was extracted with ethyl acetate. It was then evaporated at 40 °C with a rotating evaporator. From a ten-liter medium, ten grams of *Penicillium rubens* DI7S9 were obtained.

### GC-mass analysis

**Fig. 6:**
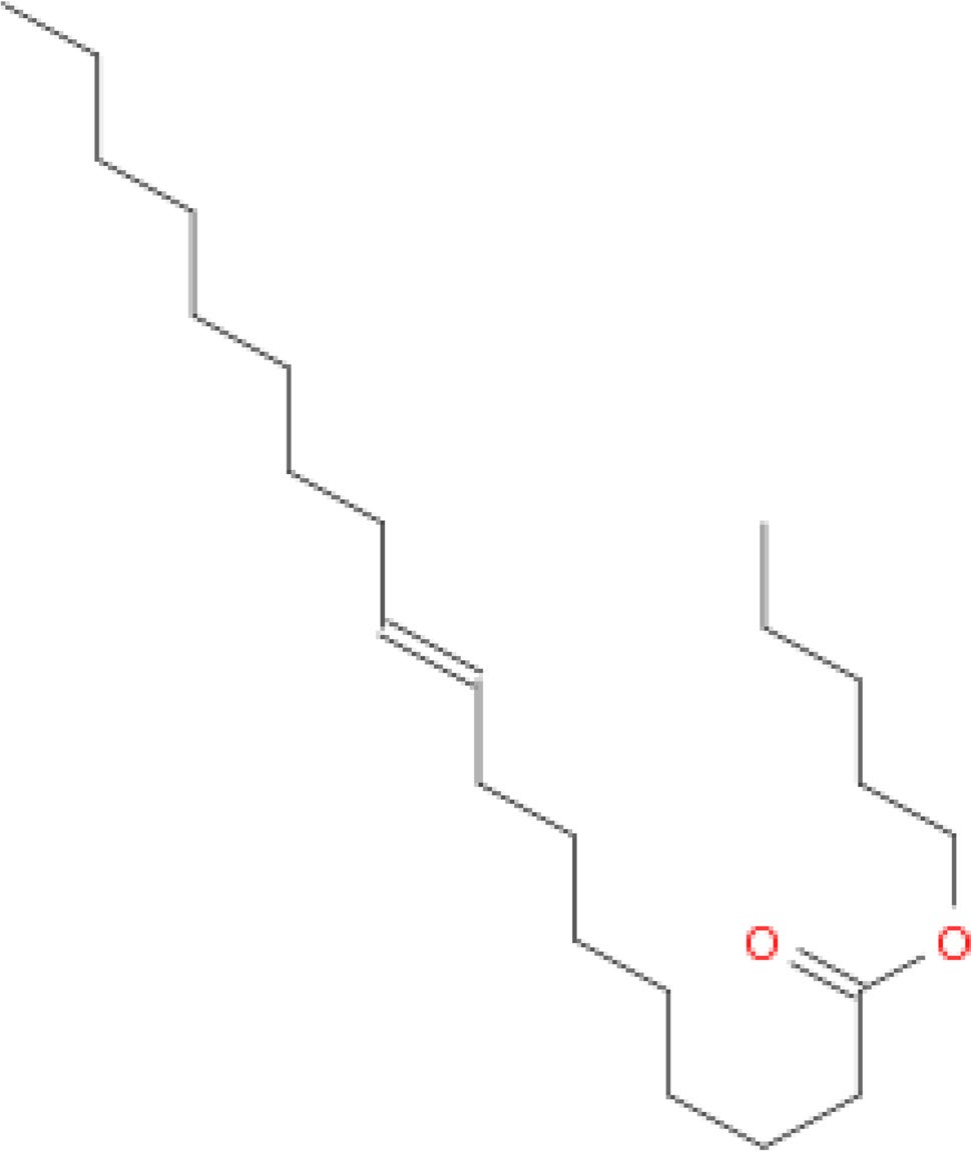
Major compound identified using GC-Mass

Gas chromatography (GC) mass analysis is a potent analytical method to identify and measure a sample’s constituents. GC mass analysis provides important information about the chemical makeup of the crude extract obtained from *Penicillium rubens* DI7S9. GC mass analysis can identify and quantify these chemicals to ascertain the crude extract’s chemical composition supplementary Figure (3). This data may benefit pharmaceutical drug development, natural product quality control, and research. The crude extract from *Penicillium rubens* DI7S9 may include bioactive chemicals, and GC mass analysis can define its chemical makeup [29]. The primary ingredients of the fungal extract include trans-9-octadecenoic acid, pentyl ester (C23H44O2), and molecular weight 352, according to GC mass analysis. This means this chemical makes up much of the extract (Fig. 6).

GC–MS analysis of the crude extract of Penicillium rubens DI7S9 revealed multiple peaks corresponding to different metabolites, as shown in the GC chromatogram (Fig. 6). The major compound identified was trans-9-octadecenoic acid, pentyl ester (C23H44O2; MW 352), which represented the predominant peak in the chromatogram. The presence of several minor peaks indicates the occurrence of additional bioactive constituents within the fungal extract. These findings confirm the chemical complexity of the metabolites produced by the marine-derived fungus.

### Cytotoxic effect of the *Penicillium rubens* DI7S9 crude extract

**Fig. 7:**
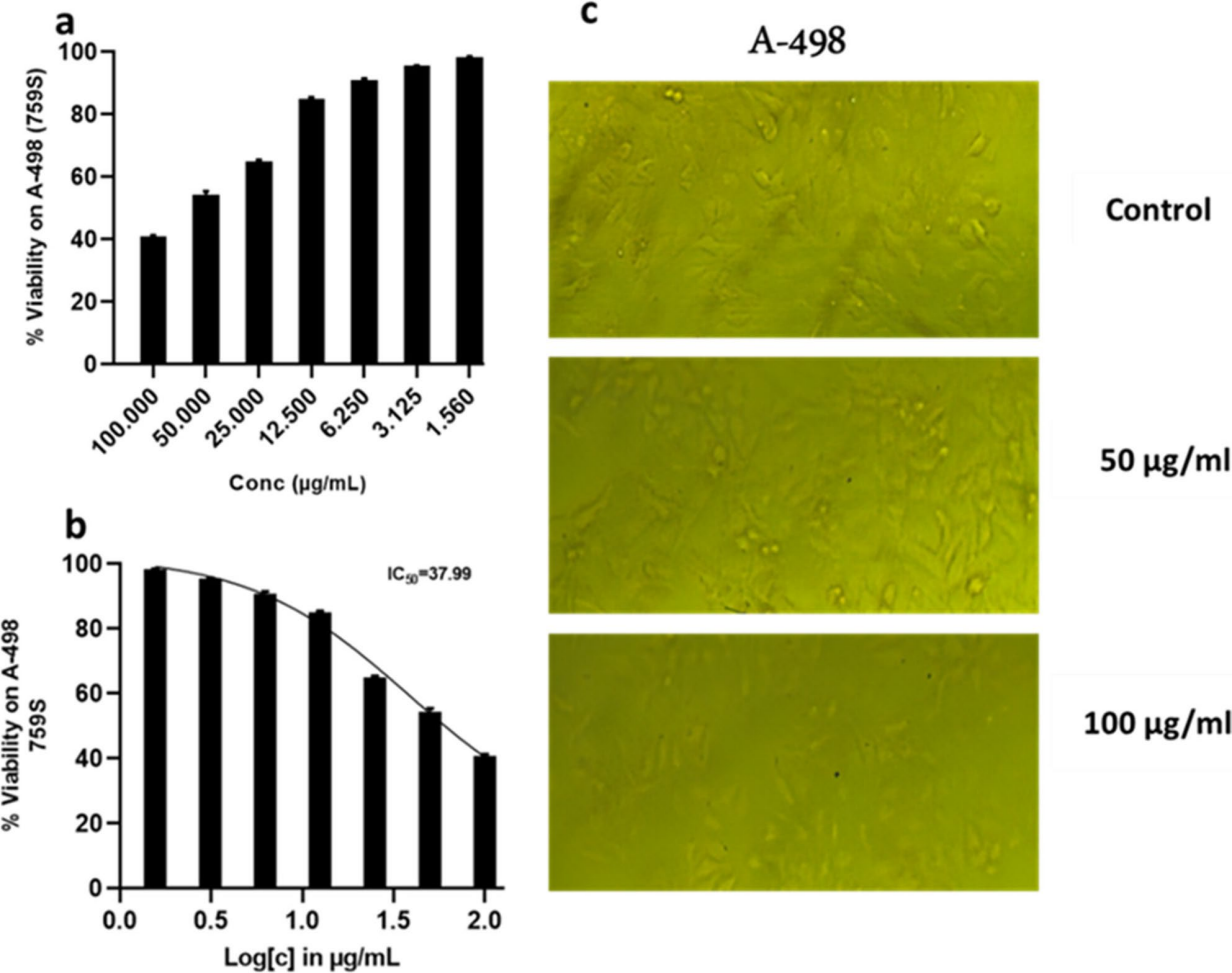
Crude extract's anticancer activity and IC50 against A-498 while (**a**) viability and (**b**) cell morphology after treatments

**Fig. 8:**
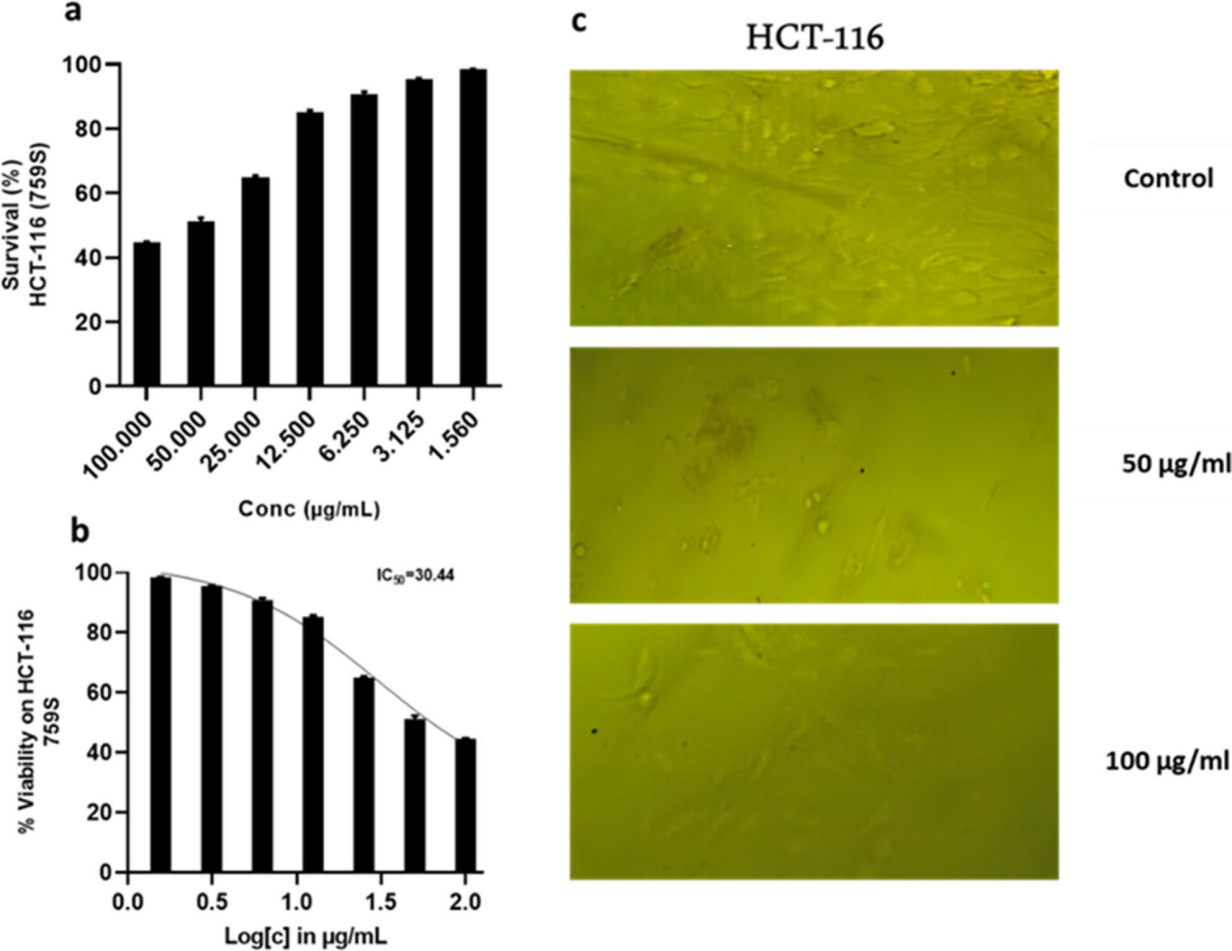
Crude extract’s anticancer activity and IC50 against HCT-116

**Fig. 9:**
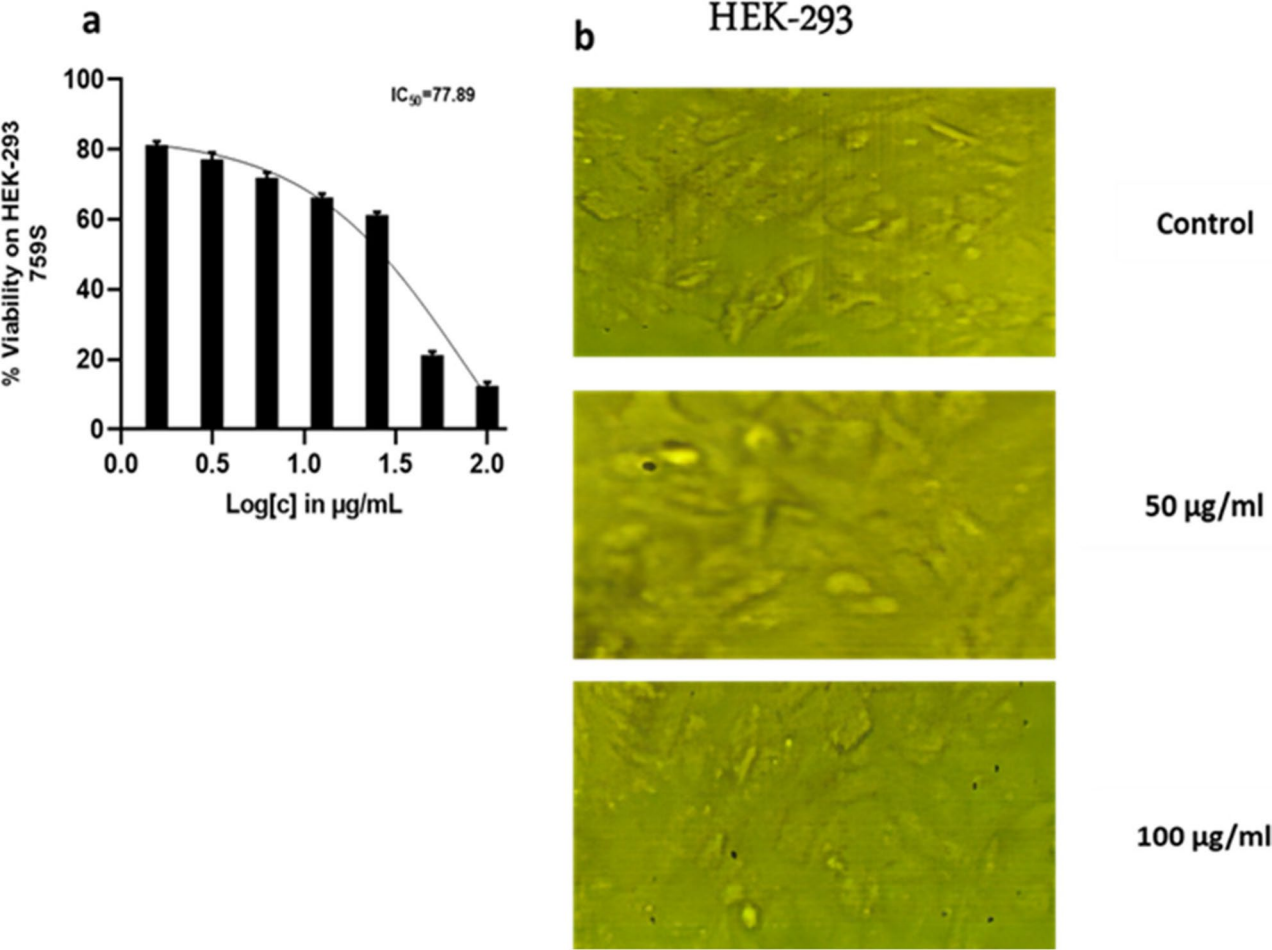
Crude extract’s anticancer activity and IC50 against HEK-293

Crude extract of *Penicillium rubens* DI7S9 has anticancer properties. The fungal crude extract’s potential as a therapeutic agent for the treatment of cancer is demonstrated by the results of its anticancer activity research against three different cell lines, including a standard cell line (HEK-293) and two cancer cell lines (HCT-116 and A-498) (Figs. 7, 8 and 9). The variations in IC_50_ values between the cancer cell lines (HCT-116 and A-498) and the standard cell line (HEK-293) suggest that the fungus crude extract may be selectively cytotoxic. The extract’s lower cytotoxic effect on healthy cells, as demonstrated by the higher IC_50_ value of 77.89 for the standard cell line, suggests it may be a safer option for cancer treatment. Selective cytotoxicity, which reduces the risk of harming healthy tissues and the side effects of conventional cancer treatments, is a crucial characteristic of anticancer medications. Meanwhile, lower IC_50_ values for the cancer cell lines HCT-116 (30.44) and A-498 (37.99) demonstrate the fungal crude extract’s strong anticancer efficacy against these specific cancer types. The extract may have a bright future as a potent anticancer treatment, given its capacity to inhibit cancer cell growth and produce cytotoxicity at such low concentrations.

Furthermore, the distinct IC_50_ values between A-498 and HCT-116 show that the two cancer cell lines respond differently to the fungus crude extract. The results indicate that the fungal crude extract selectively kills cancer cells while having minimal effect on healthy cells, suggesting a promising anticancer effect. These results indicate that it may be a lead compound in creating specific anticancer treatments. More research is necessary to completely comprehend the mechanisms of action and maximize its efficacy for prospective clinical applications [6].

### ADME-physiochemical properties

The compound “9-Octadecenoic acid, pentyl ester” possesses ADME-physiochemical qualities that imply it is a large aliphatic molecule with high structural flexibility. Since it lacks aromatic heavy atoms and has a large number of sp3 hybridized carbons, it is unlikely to have aromatic characteristics. The compound’s hydrogen bond acceptors, total polar surface area (TPSA), and molar refractivity suggest bioactivity and particular intermolecular interactions. The compound’s bulk and complexity are revealed by its 310.51 g/mol molecular weight.

**Fig. 10:**
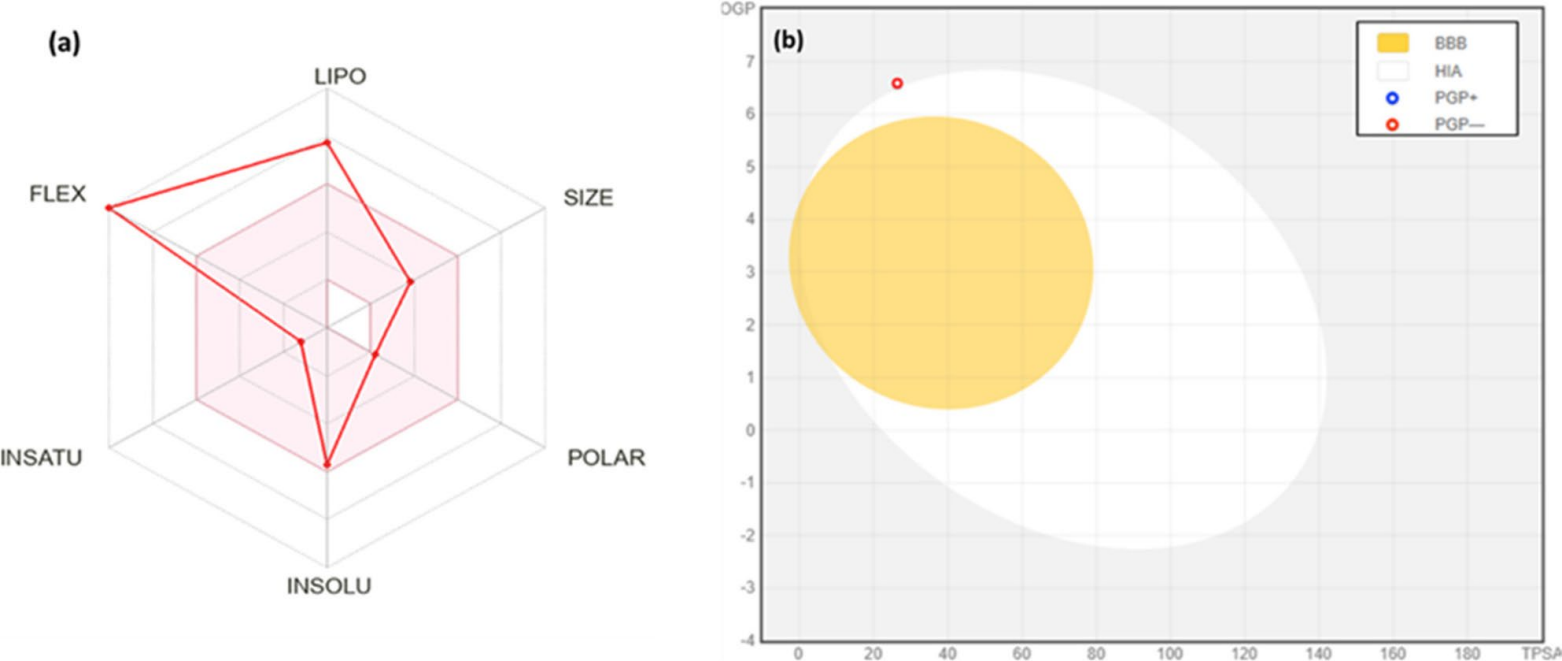
**A **Bioavailability Radar plot of the obtained compound and (**b**) BOILED-EGG plots for C_1_ and C_2_

The SwissADME web server assessed “9-Octadecenoic acid, pentyl ester” ADME physicochemical properties [30]. Drug-likeness criteria were used to evaluate pure substances (Table 3). The compound violated Lipinski’s criteria with one MLOGP (> 4.15), Rotor (> 10), and WLOGP (> 5.6). Veber’s regulations were ignored. Fig. 10a shows a Bioavailability Radar map that quickly assessed the compound’s drug-likeness. This graphic shows size, polarity, lipophilicity, solubility, flexibility, and saturation. Except for flexibility and lipophilicity, the pink plot region has the best parameter values.

**Table 3:** Physicochemical parameters of the obtained compound related to ADME

parameters for predictive models		Compound 1
Physical and Chemical Features	Weight in molecules	310.51
	Fraction Csp3	0.85
	Rotatable bonds	0
	H-bond acceptors	2
	H-bond donors	0
	Molar Refractivity	99.06
	Topological polar surface area (TPSA)	26.30 Å2
Lipophilicity	log Po/w (XLOGP3)	8.03
	log Po/w (WLOGP)	6.59
	log Po/w (MLOGP)	5.03
Solubility	log S (ESOL)	− 5.70
	Solubility	6.17e-04 mg/ml ; 1.99e-06 mol/l L
	Class	Poorly soluble
Druglikeness	Lipinski (RO5)	Yes; 1 violation: MLOGP > 4.15
	Ghose	No; 1 violation: WLOGP > 5.6
	Veber	No; 1 violation: Rotors > 10
	Bioavailability Score	0.55
Leadlikness	Rule of three (RO3)	
	Synthetic accessibility	3.34
Pharmacokinetics Parameters	GI (HIA) absorption	Low
	BBB permeant	No
	P-gp substrate	No
	CYP1A2 inhibitor	Yes
	CYP2C19 inhibitor	No
	CYP2C9 inhibitor	No
	CYP2D6 inhibitor	No
	CYP3A4 inhibitor	No
	log Kp (skin permeation: cm/s)	− 2.49 cm/s

Lipophilicity—a compound’s cell membrane permeability—is also important [31, 32]. Log Po/w values for the examined chemical surpassed 5, suggesting restricted cell membrane permeability and absorption. Solubility is crucial to medication absorption in any formulation [30]. ESOL topological model says molecule isn’t highly soluble. One infraction disqualified the compound from medical chemistry and lead toxicity requirements. The isolated molecule has modest synthetic accessibility (3.67) according to the SAscore, which measures fragment similarity and complexity penalties.

Log Po/w is the partition coefficient of n-octanol in water, while log S is the decimal logarithm of molar solubility. Range of lipinski (RO_5_) criteria: MW 500 has 5 H-bond donors and 10 acceptors. The Ghose filter has a log Po/w range of 0.4 to + 5.6, MR 40 to 130, MW 180 to 480, and atom count 20 to 70. RB 10 and TPSA 1402 have different Veber rules. Three H-bond donors, three acceptors, and three RBs are required for RO_3_ from XLOGP3 3.5 to MW 350. The synthetic accessibility scale has 10 levels, from one (very basic) to ten.

The BOILED-Egg model in Fig. 10b was inspired by Daina [30]. WLOGP and TPSA are compared in this model. The BOILED-Egg model helps explain medication penetration through intestinal membranes or the brain.

It was interesting that the substance being investigated exhibited low GI absorption. As seen by the red dots in the model, these compounds were non-P-gp substrates and only partly entered the BBB. We assessed skin permeability using Potts and Guy’s approach [31]. Significant was the compound’s log (Kp) value of -2.49 cm/s. Compounds with a negative log Kp value have decreased skin permeability.

## Conclusion

Finally, the investigation of bioactive metabolites produced by sponge-associated fungi highlights their significant potential in drug discovery and biomedical research. In the present study, Penicillium rubens DI7S9, isolated from a marine sponge, produced bioactive secondary metabolites exhibiting antimicrobial activity and selective cytotoxicity against cancer cell lines (HCT-116 and A-498) with reduced toxicity toward normal cells (HEK-293).

The observed selective cytotoxic activity represents an important characteristic for the development of targeted anticancer therapies with minimized adverse effects on normal tissues. Moreover, the identification of 9-Octadecenoic acid, pentyl ester as a major constituent of the fungal crude extract suggests that this compound may serve as a promising lead molecule for future drug development and therapeutic applications. However, further studies are required to elucidate its mechanisms of action and to evaluate its efficacy and safety in vivo.

## Supplementary Information

Supplementary Material 1.

## Data Availability

The datasets generated and/or analysed during the current study are available in the NCBI repository, [https://www.ncbi.nlm.nih.gov/nuccore/OP709283.1/] with accession number OP709283.1.
