# Supplementary material for: Bioactive metabolites from marine fungal sources: evaluation of antimicrobial and cytotoxic activities
Source: BMC Microbiol. 2026 Apr 6;26:353. doi: 10.1186/s12866-026-04962-4 (PMC13081409; doi:10.1186/s12866-026-04962-4)

Supplementary Table 1: Sample data from Hurghada

| **Locations** | **Sponge code** |
| --- | --- |
| **Location 1** | 1A |
|  | 2A |
|  | 3A |
| **Location 2** | 4A |
|  | 5A |
|  | 6A |


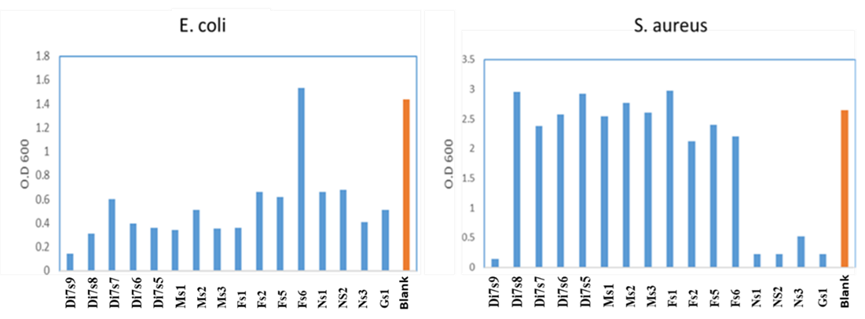


Supplementary Fig. 1: Fungal extract's antibacterial activity against S. aureus and E. coli


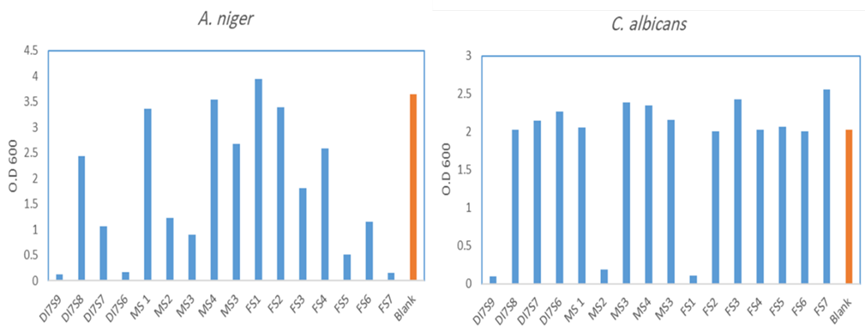


Supplementary Fig.2: Fungal extract's antifungal activity against Candida albicans and A. niger

Supplementary Fig 3. GC chromatogram of the fungal crude extract


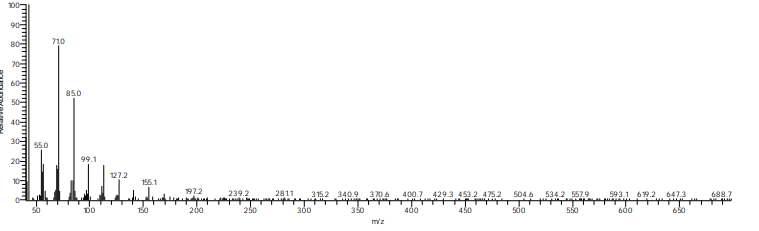

Supplement: Supplementary file 1 — Supplementary Material 1. [file 12866_2026_4962_MOESM1_ESM.docx]
